# Development of a High-Throughput Platform for Quantitation of Histone Modifications on a New QTOF Instrument

**DOI:** 10.1016/j.mcpro.2024.100897

**Published:** 2024-12-19

**Authors:** Emily Zahn, Yixuan Xie, Xingyu Liu, Rashmi Karki, Richard M. Searfoss, Francisca N. de Luna Vitorino, Joanna K. Lempiäinen, Joanna Gongora, Zongtao Lin, Chenfeng Zhao, Zuo-Fei Yuan, Benjamin A. Garcia

**Affiliations:** 1 Department of Biochemistry and Molecular Biophysics, Washington University School of Medicine, St Louis, Missouri, United States; 2 State Key Laboratory of Genetic Engineering, Greater Bay Area Institute of Precision Medicine (Guangzhou), School of Life Sciences and Institutes of Biomedical Sciences, Fudan University, Shanghai, China; 3 Department of Computer Science and Engineering, Washington University in St Louis, St Louis, Missouri, United States; 4 Center for Proteomics and Metabolomics, St Jude Children’s Research Hospital, Memphis, Tennessee, United States

**Keywords:** histone, DIA, post-translational modification, mass spectrometry, epigenetics

## Abstract

Histone post-translational modifications (PTMs) regulate gene expression patterns through epigenetic mechanisms. The five histone proteins (H1, H2A, H2B, H3, and H4) are extensively modified, with over 75 distinct modification types spanning more than 200 sites. Despite strong advances in mass spectrometry (MS)–based approaches, identification and quantification of modified histone peptides remains challenging because of factors, such as isobaric peptides, pseudo-isobaric PTMs, and low stoichiometry of certain marks. Here, we describe the development of a new high-throughput method to identify and quantify over 150 modified histone peptides by LC–MS. Fast gradient microflow liquid chromatography and variable window sequential windows acquisition of all theoretical spectra data-independent acquisition on a new quadrupole time-of-flight platform is compared to a previous method using nanoflow LC–MS on an Orbitrap hybrid. Histones extracted from cells treated with either a histone deacetylase inhibitor or transforming growth factor-beta 1 were analyzed by data-independent acquisition on two mass spectrometers: an Orbitrap Exploris 240 with a 55-min nanoflow LC gradient and the SCIEX ZenoTOF 7600 with a 10-min microflow gradient. To demonstrate the reproducibility and speed advantage of the method, 100 consecutive injections of one sample were performed in less than 2 days on the quadrupole time-of-flight platform. The result is the comprehensive characterization of histone PTMs achieved in less than 20 min of total run time using only 200 ng of sample. Results for drug-treated histone samples are comparable to those produced by the previous method and can be achieved using less than one-third of the instrument time.

## Highlights

• New high-throughput LC-MS method identifies and quantifies histone PTMs in 20 min.
• New computational tools enable large-scale histone modification analysis.
• Results for drug-treated samples comparable to previous method in one-third the time.
• Ultrafast microflow gradient and SWATH DIA LC-MS platform on the ZenoTOF 7600.

## Graphical Abstract

**Figure:**
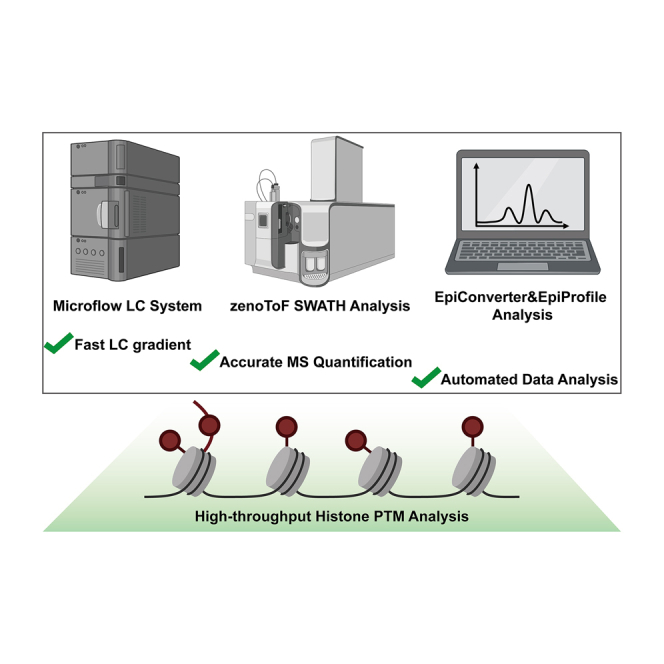

## In Brief

Here, we describe a novel and high-throughput platform for the analysis of histone post-translational modifications by LC–MS. New computational tools are introduced to enable large-scale identification and quantitation of modified histone peptides. Fast gradient microflow liquid chromatography and variable window SWATH data-independent acquisition on a new quadrupole time-of-flight platform yields comprehensive analysis of histone post-translational modifications in 20 min. Results for drug-treated histone samples are shown to be comparable to previous methods and can be achieved in one-third the instrument time.

Histones are one of the most abundant proteins involved in genomic regulation and the proper packaging of DNA into nucleosomes and further into chromatin. They are closely associated with a host of enzymes that modulate a variety of biological processes, including chromatin condensation, transcription, and DNA repair (1). Many of these enzymes function by either adding or removing post-translational modifications (PTMs) to the histones (known as writers and erasers). Several proteins also interact with the modifications, known as readers (2, 3). The pattern of PTMs that each histone carries ultimately dictates the function of the interactors at each nucleosome and genomic region, as well as the structure of the chromatin locally, at interchromosomal interactions, and over the stages of mitosis (4, 5, 6, 7).

Given the broad range of processes that involve histones, it is no surprise that there are hundreds of annotated PTMs across multiple histone isoforms, creating what is termed the “histone code” (8, 9). The histone code is a dynamic and tightly regulated process across development and in response to cellular stimuli. The most widely recognized histone modifications are lysine methylation and acetylation, both of which play major roles in DNA binding, gene regulation, and chromosomal compaction (10, 11). It is classically thought that methylation represents genomic areas where genes are silenced and is enriched in heterochromatin, and acetylation represents genomic areas that are transcriptionally active and is enriched in euchromatin. Other histone marks such as H3K4me3 are found in regions of high gene expression and transcription start sites (12). New histone modifications are still being discovered, as evident by the observation of a novel histone modification, methylacetyllysine (Kacme), in which a single lysine residue harbors both a methyl group and an acetyl group on its epsilon amine (13). This new and unique modification was further found to be associated with promoter regions of actively transcribed genes. With so much complexity contained within the histone code, represented by the exponential number of combinations of PTMs and histone isoforms, there is still plenty that is not understood about the context of each modification and their co-occurrences across the entire epigenetic landscape (14, 15). As such, there are great efforts to learn about how different modifications interact across the genome and even still more modifications to discover.

Histone PTM dysregulation, mutations, and aberrations in epigenetic machinery have already been implicated in several diseases ranging from developmental disorders, metabolic diseases, and rare cancers like malignant peripheral nerve sheath tumors and lung adenocarcinoma (16, 17, 18, 19, 20). With such clinically relevant phenotypes, both histones and histone readers have been the subject drug targets, including histone deacetylase (HDAC) inhibitors (HDACis) and poly-ADP-ribosylation inhibitors (21, 22, 23, 24, 25). While these examples have had some success in clinical applications, little is known about what readers and erasers they target, and how broadly they alter the epigenetic landscape is still under study.

To date, the most effective and accurate way to study histone modifications and epigenetic aberrations is with mass spectrometry (MS). MS remains the only approach that is sensitive enough to differentiate between isobaric and coeluting modifications, as well as quantify hundreds of modifications in a single experiment in an extremely high-throughput manner compared with other approaches such as antibody detection (26, 27, 28, 29). Classically, the study of histone modifications by MS has been performed on Orbitrap instruments using long gradients (>45 min) at nanoliter flow rates with label-free quantitation and data-independent acquisition (DIA) analysis to capture low abundant marks (30, 31). This was primarily because of the Orbitrap having the highest sensitivity to identify and quantify low abundant species compared with the low sensitivity of time-of-flight (TOF) instruments (32). However, as MS technology has improved, particularly in addressing the sensitivity issue of TOF, the application of TOF instruments has become more appealing with faster scan speeds and better duty cycle utilization compared with Orbitraps (33, 34).

The Sciex ZenoTOF 7600 is the latest generation of TOF instrument, boasting scan speeds of up to 133 Hz, increased resolution of 42,000 at *m/z* 956, and drastic improvements in sensitivity enabled by the Zeno-trapping technology (35, 36). Zeno-trapping addresses the issue of ion loss during the previously constant transfer of ions from the collision cell to the TOF accelerator by adding an ion trap to the end of the collision cell. Now, a packet of fragment ions is collected and pulsed into the accelerator so that they are uniformly received in the accelerator prior to MS analysis. Zeno-trapping has allowed for a 6-20X gain in fragment ion signal and increased the duty cycle to nearly 100%, greatly improving the sensitivity of this TOF instrument (37, 38). A schematic of the zeno trap can be found (37, 38). Furthermore, the application of sequential windows acquisition of all theoretical spectra (SWATH) analysis to histone marks is also agreeable with the previous success of DIA analysis, with the prospect of optimally designing isolation windows to best target the histone marks across the chromatographic dimension.

This instrument has already proven to be extremely powerful in a sweeping number of applications. For metabolomics and lipidomics studies, the complementary fragmentation of targets by both collisionally induced dissociation and electron-activated dissociation has allowed for better identification of isomeric compounds, and the increased sensitivity gained with the Zeno trap has allowed for more sensitive assays in diseases like prostate cancer and coronavirus disease (39, 40, 41, 42, 43). Similarly, the combination of electron-activated dissociation with Zeno-trapping has allowed for the development of a novel oligonucleotide modification discovery platform as well as a quantitative RNA platform from matrices as challenging as plasma (44, 45, 46). Glycan structure and localization, a classically challenging study, has also benefited from electron-based fragmentation by improving the detection and quantitation of multiple isomeric glycoforms in a single analysis (47, 48, 49, 50). Early examples applied directly to proteomics further demonstrate the performance improvements of the ZenoTOF 7600 by allowing increased protein identification in nanogram-scale analyses of samples ranging from human kidney tissue to animal model biofluids and intact antibodies. This instrument is also designed to excel at analytical flow rates without losing quantitative sensitivity, enabled by the Zeno-trapping technology (51, 52, 53, 54, 55, 56). This exemplary showcase makes the argument that it would be a great improvement to the current mass spectrometric approaches for histone PTM analysis.

As the instrumentation in MS evolves and improves, it is important to systematically evaluate histone PTM analysis on any new platform and identify ways to maintain or improve existing analytical methods. Recently, we published an update to the histone propionylation protocol, which resulted in a higher throughput sample preparation methodology without sacrificing the identification and quantitation of histone PTMs (57). Here, we build on this focus of increasing throughput by turning our attention to the advantages of improved instrumentation. The new Sciex ZenoTOF 7600, with an increased scan speed and increased sensitivity enabled by the Zeno trap, has allowed us to decrease the mass spectrometer (or analytical) run time to only 20 min. Furthermore, this approach has been adapted to microflow chromatographic separation, which maintains retention time stability and separation efficiency of closely eluting peptides still without affecting identification and quantitation as would be expected from microflow chromatography (58). In addition, these new data have facilitated the need to develop further computational tools (EpiProfile 2.2) to handle this new high-throughput histone PTM data (59). Taken together, we have created a new complete histone PTM profiling pipeline that can analyze three to six times more samples per day than current standard approaches by drastically shortening our LC–MS analysis time without sacrificing data quality and quantitative accuracy.

## Experimental Procedures

### Experimental Design and Statistical Rationale

In this study, a newly developed platform for comprehensive analysis of histone PTMs using fast gradient microflow LC and variable window SWATH DIA on the ZenoTOF 7600 was compared against a previously described nanoflow LC DIA method on an Orbitrap hybrid (57). The Orbitrap method’s 3-s cycle consisted of an MS1 scan collected at 60,000 resolution followed by 35 DIA windows with a width of *m/z* 24 and 1 Da overlap. In the ZenoTOF method, the 0.761-s cycle consisted of a TOF MS followed by 42 variable width DIA windows with 1 Da overlap. Histone peptides were identified and quantitated using EpiProfile, a library-free DIA search software designed specifically for DIA analysis of tryptic digests of propionylated histone samples (59). EpiProfile 2.1 was used for Orbitrap data, and a new version of the software suitable for SCIEX data introduced here, EpiProfile 2.2, was used for the ZenoTOF data. A new generic data conversion software, EpiConverter, is also introduced to convert mzXML files into a format compatible with EpiProfile.

Histone samples from untreated human cell lines were first analyzed using both methods. Three biological replicates of histones extracted from human lung adenocarcinoma cell line NCI-H1437 were analyzed by each method. A synthetic standard peptide with properties similar to histone peptides was spiked in as previously described (57). The number of histone peptides identified, the average points across the peak, and the CVs of the peak area, peptide ratio, and retention time were compared for each method.

To demonstrate the applicability of the method to biological questions, histones extracted from cells treated with HDACis were analyzed. Three biological replicates of histones extracted from untreated HeLa cells and three biological replicates for each drug-treated condition (M344 and entinostat) were analyzed. Metaboanalyst (https://www.metaboanalyst.ca/docs/About.xhtml) was used to visualize data. During data input, no normalization, transformation, or scaling was performed. To visualize data in hierarchical clustering heatmaps, Euclidean distance measure was applied with Ward clustering method. For correlation heatmap, Pearson *r* was used for distance measure. To generate a volcano plot, a fold change threshold of two and *p* value threshold of 0.05 were considered significantly different. Principal component analysis was performed using PERMANOVA to evaluate statistical significances for biological replicates.

To demonstrate that the method could identify changes in histone modifications more subtle than those induced by treatment with an HDACi, histones extracted from cells treated with transforming growth factor beta (TGF-β) to induce epithelial–mesenchymal transition (EMT) were also analyzed using each method. Three control biological replicates of histones extracted from untreated H1437 cells and three biological replicates of TGF-β-treated samples were analyzed. A *t* test was performed, and peptides meeting a fold change threshold of 1.5 and two-tailed *p*-value threshold of 0.05 were considered significantly altered.

To demonstrate the speed and reliability of the ZenoTOF method, a single sample of histones extracted from untreated HeLa cells was injected and analyzed 100 times in 2 days. The number of histone peptides identified in each run and the CV values of the peak area, peptide ratio, and retention time of histone peptides were determined.

### Preparation of HDACi-Treated Cells

The HeLa cell line was obtained from the American Type Culture Collection. The cell line was grown at 37 °C with a 5% CO_2_ in Dulbecco’s modified Eagle’s medium supplemented with 10% fetal bovine serum, penicillin (100 U/ml), streptomycin (100 μg/ml), and 2 mM GlutaMAX Supplement (Gibco; catalog no.: 35050061). HeLa cells were plated a day earlier at a density of 1 × 10^5^ cells/10 ml in four 10-cm dishes at 37 °C. HDACis (entinostat and M344) were purchased from Selleck Chemicals as a 10 mM stock solution. The solution was then serially diluted with the culture medium to obtain the desired concentration. HeLa cells were treated with the manufacturer’s IC_50_ concentration for 24 h. The IC_50_ used for entinostat and M344 in this study is 1 μM. Each drug treatment involved three biological replicates.

Cells were trypsinized by adding ∼500 μl of trypsin and incubated at 37 °C for 5 min. About 1 ml of PBS was added to each plate, and cells were scraped into a solution before being collected and transferred to a 1.5 ml Eppendorf tube. Cells were centrifuged at 800*g* for 5 min, and the PBS was aspirated off. Cell pellets were resuspended once more with PBS to remove excess culture media, centrifuged at 800*g* for 5 min, and the PBS aspirated off. Pellets were frozen at −80 °C until further processing once more with PBS to remove excess culture media, centrifuged at 800*g* for 5 min, and the PBS aspirated off.

### Preparation of TGF-β1–Treated Cells

Human lung adenocarcinoma cell line NCI-H1437 (American Type Culture Collection) was cultured in RPMI1640 supplemented with 10% fetal bovine serum, GlutaMAX, and MEM Nonessential Amino Acids. EMT of H1437 cells was induced by treating cells with Recombinant Human TGF-β1 (R&D System) at 10 ng/ml in their regular culture medium for 3 days. Cells were harvested in PBS, and cell pellets were frozen at −80 °C until further processing.

### Histone Extraction and Propionylation

Histones were extracted from cells and propionylated following previously published protocols (57). Briefly, cells were lysed with a Nuclear Isolation Buffer. The lysate was centrifuged, and the pellet was washed to remove detergent and cytoplasmic contents. Histones were extracted from nuclei by incubation in H_2_SO_4_. After centrifugation, histones were precipitated overnight by trichloroacetic acid and then rinsed with acetone and allowed to air-dry. Histones were resuspended in Milli-Q water. Histone concentration was determined by detergent-compatible assay.

Extracted histones were resuspended in 50 mM ammonium bicarbonate, and a spike-in histone standard peptide was added (57). The pH was adjusted to pH 8.0 with ammonium hydroxide and formic acid. A 25% solution of propionic anhydride in acetonitrile (ACN) was added to each sample at a 1:2 ratio. About 7 μl of ammonium hydroxide was added to prevent acidification of the solution. Samples were vacuum centrifuged to dryness. Samples were resuspended in 50 mM ammonium bicarbonate. Propionylation was repeated to ensure complete derivatization. Samples were resuspended to a 1 μg/μl concentration and digested at room temperature overnight with trypsin at a 1:50 enzyme:sample ratio. After vacuum centrifugation to quench the digestion, samples were resuspended in 50 mM ammonium bicarbonate, and the propionylation protocol was repeated a final time to derivatize the N termini of the digested peptides.

Samples were desalted by in-house stage tips prepared using a previously described protocol (57). Stage tips were equilibrated twice with 80 μl of 80% ACN and twice with 80 μl of 0.1% TFA with centrifugation at 2000*g* for 1 min following each step. Peptides were resuspended in 100 μl of 0.1% TFA and loaded onto the stage tips with centrifugation at 1000*g* for 1 min. Stage tips were rinsed twice with 80 μl of 0.1% TFA with centrifugation at 2000*g* for 1 min. Peptides were eluted with two rounds of 80 μl of 80% ACN with centrifugation at 2000*g* for 1 min. Samples were vacuum centrifuged to dryness and stored at −20 °C until further analysis.

### LC–MS Analysis

Samples were analyzed on an M5 MicroLC (SCIEX) or an ACQUITY UPLC M-Class (Waters) coupled to a ZenoTOF 7600 mass spectrometer (SCIEX). An OptiFlow TurboV ion source was used with a microflow probe. Mobile phase A was 0.1% formic acid, and mobile phase B was 0.1% formic acid in ACN. Samples were run by direct injection on a Kinetex XB C18, 100 Å, 2.6 μm, 0.3 × 150 mm column (Phenomenex) at a flow rate of 10 μl/min. About 200 ng of the sample was injected for each analysis. The mass spectrometer was operated in ZenoSWATH DIA mode with collisionally induced dissociation fragmentation. The TOF MS scan range was *m/z* 120 to 1400. Dynamic collision energy was enabled and ranged from 14 to 46 V. The LC gradient and SWATH isolation scheme were optimized for either a 10-min linear gradient from 5 to 32% solvent B or a 5-min linear gradient from 5 to 30% solvent B. TOF MS accumulation time was 100 ms, and MS2 accumulation time was 10 ms.

Samples were also analyzed on a Vanquish Neo UHPLC (Thermo Fisher Scientific) coupled to an Orbitrap Exploris 240 (Thermo Fisher Scientific) or an Orbitrap Exploris 240 (Thermo Fisher Scientific) coupled to an Omnitrap platform (Fasmatech), operated in Orbitrap-only mode. Solvent A was 0.1% formic acid in water, and solvent B was 0.1% formic acid in ACN. The LC gradient consisted of 2 to 32% solvent B in 48 min followed by 32 to 42% solvent B in 7 min. The LC was operated in trap-and-elute mode using a PepMap Neo C18, 100 Å, 5 μm, 0.3 × 5 mm Trap Cartridge (Thermo Fisher Scientific) and an Easy-Spray PepMap Neo C18, 100 Å, 2 μm, 75 μm × 150 mm column (Thermo Fisher Scientific). The flow rate was 300 nl/min. About 1 μg of sample was injected for each analysis. The mass spectrometer was operated in DIA mode with an MS1 scan range of *m/z* 290 to 1115 at 60,000 resolution and an MS2 resolution of 15,000. About 35 DIA windows with a width of *m/z* 24 and center masses from *m/z* 307 to 1089 with 1 Da overlaps were used. Higher energy collision dissociation collision energy was 30%.

### Data Analysis

Raw files produced by the Exploris 240 were searched with EpiProfile 2.1 to identify and quantitate histone peptides (59, 60). Wiff files produced by the ZenoTOF 7600 were converted to mxXML using a new software we developed for this purpose (EpiConverter) and searched with an updated version of EpiProfile. Data were further processed in Excel and Skyline (MacCoss lab, University of Washington, Seattle) (61). Skyline 24.1.0.199 was used to perform the analysis. The list of all peptides searched by EpiProfile was added to Skyline using the proteomics interface. Peptide settings included enzyme trypsin derivatization [R|P] with 0 miss cleavages and no retention time predictor. Modifications were manually added to each peptide. For the transition settings, precursor and product ion mass were set as monoisotopic, with filters for precursor charges 2 to 4, product ion charge 1, and ion types y, b, and p. A minimum of *m/z* 50 and a maximum of *m/z* 1500 with a method-match tolerance of *m/z* 0.055 were used for the instrument settings. Full-scan MS1 filtering was as follows: isotope peaks included—count, precursor mass analyzer—centroid, peaks—3, mass accuracy—10 ppm; MS/MS filtering: acquisition method—DIA, product mass analyzer—centroided, mass accuracy 20 ppm, isolation scheme—results only; and retention time filtering including all matching scans.

## Results and Discussion

### Establishment of SWATH Analysis on the ZenoTOF 7600 for High-Throughput Histone PTM Analysis

**Fig. 1:**
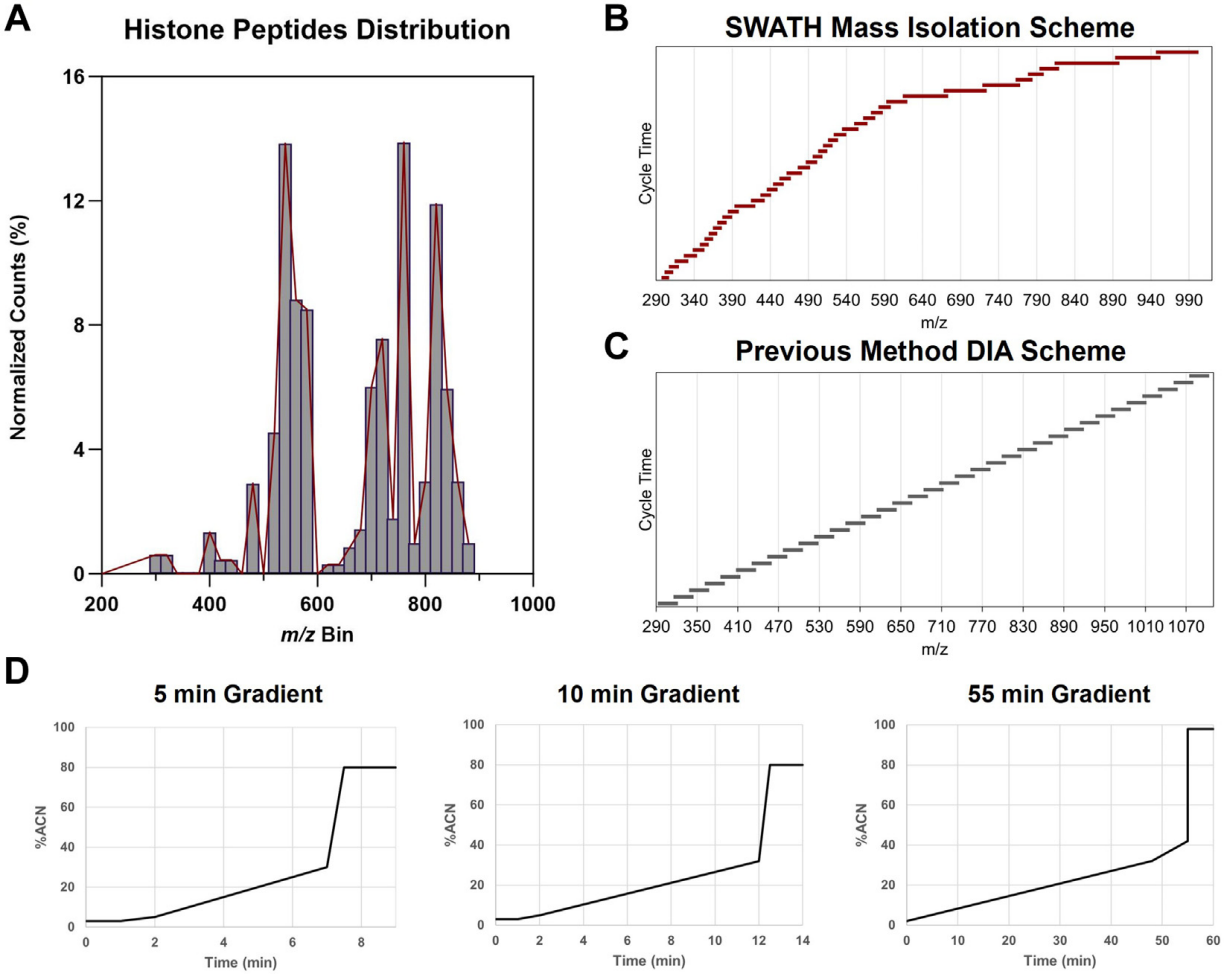
**SWATH DIA scheme and LC gradients.***A*, distribution of histone peptides across the mass range. *B*, SWATH mass isolation scheme for histone peptides consisting of 42 variable width windows. *C*, DIA scheme previously utilized on the Orbitrap Exploris, consisting of 35 fixed-width windows with 1 Da overlap. *D*, 5-min and 10-min linear microflow gradients developed for SWATH DIA on the ZenoTOF and 55-min nanoflow gradient used for analysis on the Orbitrap Exploris. DIA, data-independent acquisition; SWATH, sequential windows acquisition of all theoretical spectra.

To find the optimal SWATH parameters, we meticulously optimized the SWATH windows and LC gradients based on the unique features of histone peptides. As SWATH analysis fragments all precursor ions within a defined isolation window, generating complicated fragment product ion spectra from multiplexed precursors, it is critical to optimize the mass window scheme for the histone PTM analysis. Therefore, we prescanned and determined the general histone PTM distributions over our normal *m/z* range. As shown in Figure 1*A*, we found that the diverse modified histone peptides allocate to a wide mass-to-charge range of *m/z* 300 to 900. After confirming the precursor window range, we evaluated the number of SWATH windows we might utilize, including 40, 60, and 80 windows. In each case, a variable window scheme tailored to the distribution of histone peptides across the *m/z* range was generated using the SWATH Acquisition Variable Window Calculator from SCIEX. Windows were narrower in areas of the scan range with more histone peptides present to be identified and wider in areas of the scan range where fewer histone peptides are found. Concurrently, we also considered the overall cycle of scans by adjusting the MS2 accumulation time to 10, 20, and 30 ms, controlling the total scan time. As a result, we observed that 42 SWATH windows and 10-ms MS2 accumulation time provided superior results in generating limited interfering ions and accurate identification within the total scan time of 0.76 s. The representative scan scheme of SWATH windowing and detailed parameters are shown in Figure 1*B* and Supplemental Table S1. The DIA scheme and detailed parameters for the previous method developed on the Orbitrap are provided in Figure 1*C* and Supplemental Table S2.

Furthermore, we investigated the LC gradient for the histone analysis. The increased speed of the ZenoTOF compared with Orbitrap instruments allows the use of shorter gradients. To take advantage of this increase in speed, we developed an ultrafast microflow gradient for the separation of histone peptides. Previous data demonstrated that histone peptides are well separated in nanoflow schemes by gradients consisting of 2 to 32% ACN in 48 min followed by an increase to 42% ACN in 7 min (57). Adapting this method, we tested microflow gradients including 5 to 32% B in 10 min followed by an increase to 45% B in 4 min, a 10-min linear gradient from 5 to 32% B, and a 10-min linear gradient from 5 to 45% B, and a 5-min linear gradient from 5 to 30% B. Flow rates of 5 μl/min and 10 μl/min were compared for the various gradients, and column washing and equilibration were optimized to minimize total run time without sacrificing robust and reproducible chromatography. Ultimately, a linear gradient of 5 to 32% B in 10 min with a total run time of 18 min was found to be optimal for separating histone peptides and supporting ultrafast comprehensive analysis of histone PTMs with ZenoSWATH DIA. A shorter gradient consisting of 5 to 30% B in 5 min with a total run time of 11 min was also selected as being suitable for the analysis of histone PTMs in applications that may prioritize time savings over maximizing the depth of information obtained, such as screens of large-batch cohorts or analyses concerning large changes in the distribution of histone PTMs. The LC gradient curves and detailed parameters are shown in Figure 1 and Supplemental Tables S1–S6.

**Fig. 2:**
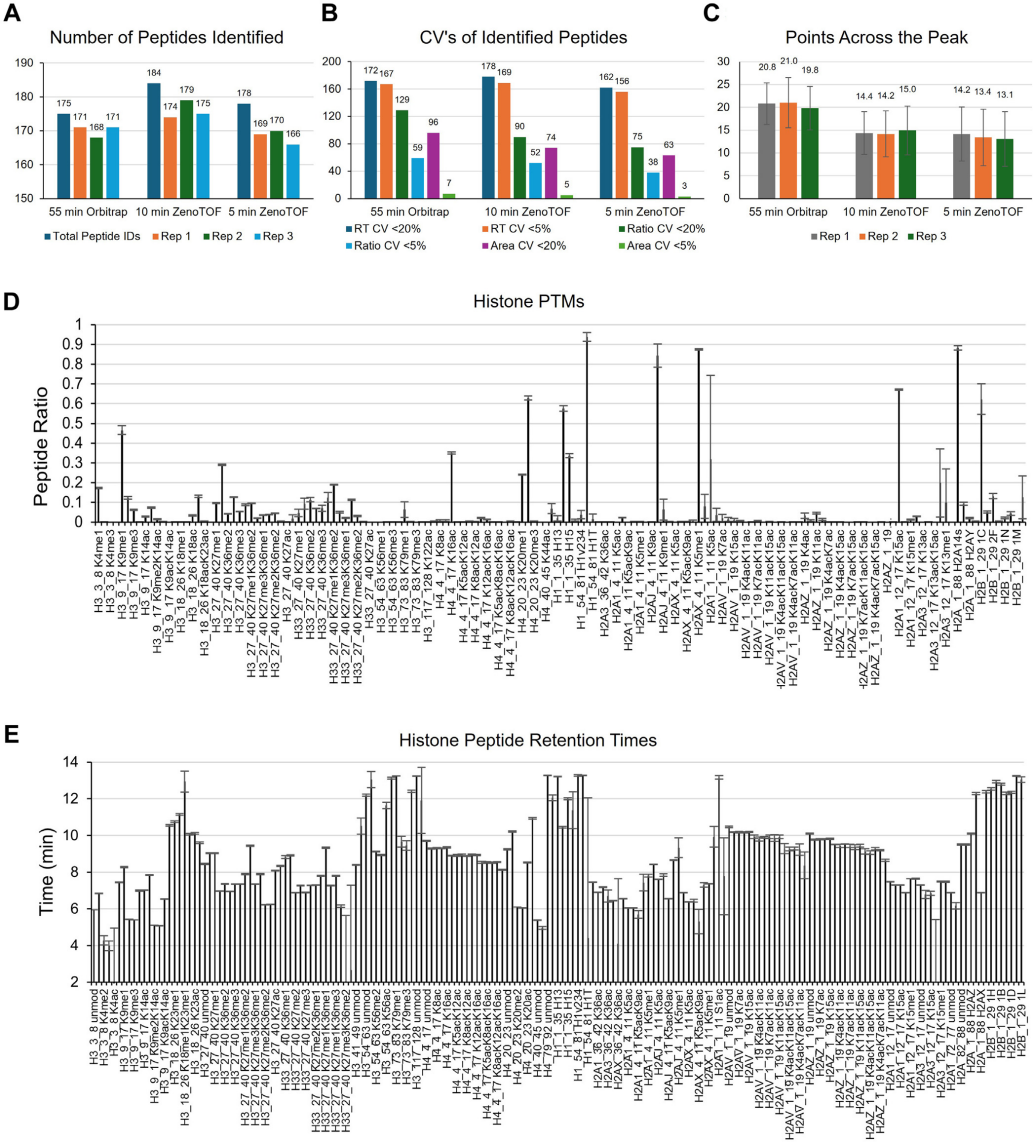
**Identification and quantification of histone peptides in H1437 cells with ZenoSWATH DIA and fast microflow gradient.***A*, number of peptides identified by EpiProfile 2.2 in three biological replicates analyzed with the 55-min nanoflow gradient on the Orbitrap Exploris or the 5- or 10-min microflow gradient on the ZenoTOF. *B*, number of peptides identified by EpiProfile 2.2 in each method with CV below 20% and below 5% for peptide retention time, peak area, and peptide ratio. *C*, average points across the peak for histone peptides in each of the three biological replicates by each method. Values were extracted using Skyline. *D*, modified histone peptides identified with the 10-min gradient ZenoTOF method, with average observed peptide ratios. *E*, average retention time of histone peptides observed using the 10-min microflow gradient. DIA, data-independent acquisition.

The advantages of the newly developed platform are demonstrated by comparing the analysis of three biological replicates of histone peptides extracted from H1437 cells on the ZenoTOF and the Orbitrap Exploris. The total number of histone peptides identified by both the 5-min and 10-min methods is higher than that achieved by the Orbitrap analysis, with 178 and 184 peptides identified respectively, as shown in Figure 2*A*. The observed CV values for peptide ratio and mass area are comparable between the two methods. Notably, the stability of histone peptide retention times across runs is also comparable to that of the longer nanoflow method, with the majority of identified peptides having retention time CV values below 5% (Fig. 2*B*). In addition, the speed of the ZenoTOF combined with the customized SWATH windows generates a sufficient number of points across the peak for accurate quantitation even with the greatly reduced run time and reduced LC peak widths (Fig. 2*C*). Comprehensive characterization of histone peptides that matched or exceeded that of the previous method was achieved in one-third of the instrument time.

### Development of Automated Computational Data Analysis Workflow

Identification and quantification of histone peptides are particularly challenging because of the presence of isobaric peptides, different pseudoisobaric PTMs, and low abundance of species. For example, acetylation can be found in four different lysine residues within the same H4 peptide, GKGGKGLGKGGAKR. Meanwhile, two common histone modifications, trimethylation and acetylation, have similar mass shifts, 42.047 Da and 42.011 Da, respectively. With the example shown previously, the data can be integrated manually using software such as Skyline (61); however, the manual interpretation of different histone modifications is time consuming, and it is not possible for a large-scale batch cohort. This is a limitation for any quantitative proteomics approach, as little to no automated computational resources to aid in data analysis are crucially limiting.

One challenge in adapting the histone analysis platform to the Sciex ZenoTOF 7600 was in data processing. Our previous histone data analysis software, EpiProfile 2.1, was designed primarily for RAW files and for extended LC gradients to search for isobaric peptides (59). To meet the need for computational tools that enable large-scale identification and quantification in automated form with this new high-throughput method, we developed a generic data conversion software, EpiConverter, to format the “mzXML” file into “ms1” and “ms2” files, which can be analyzed with our EpiProfile software. Specifically, EpiConverter was built to properly convert Sciex WIFF files to a usable.mzML structure for EpiProfile. We next integrated these converted data with our EpiProfile (version 2.1), as the generated “ms1” and “ms2” files can be examined to resolve the comprehensive histone PTM information, including the isobaric histone peptides, low-abundant PTMs, different histone mutations, different derivatization strategies, and histone sequences from various organisms (59). Importantly, the workflow can be applied for both label-free and isotopic labeling strategies, providing more universal applications for investigating the dynamics of histone codes (59). However, we did encounter another challenge, as we observed incorrect assignments or lack of identification of common peptides because of increased elution overlap as a result of shortened analytical gradients. This led to the development of an updated EpiProfile version, which was improved and optimized to be able to handle the ultrafast gradients and still accurately identify and quantify the histone marks.

**Fig 3:**
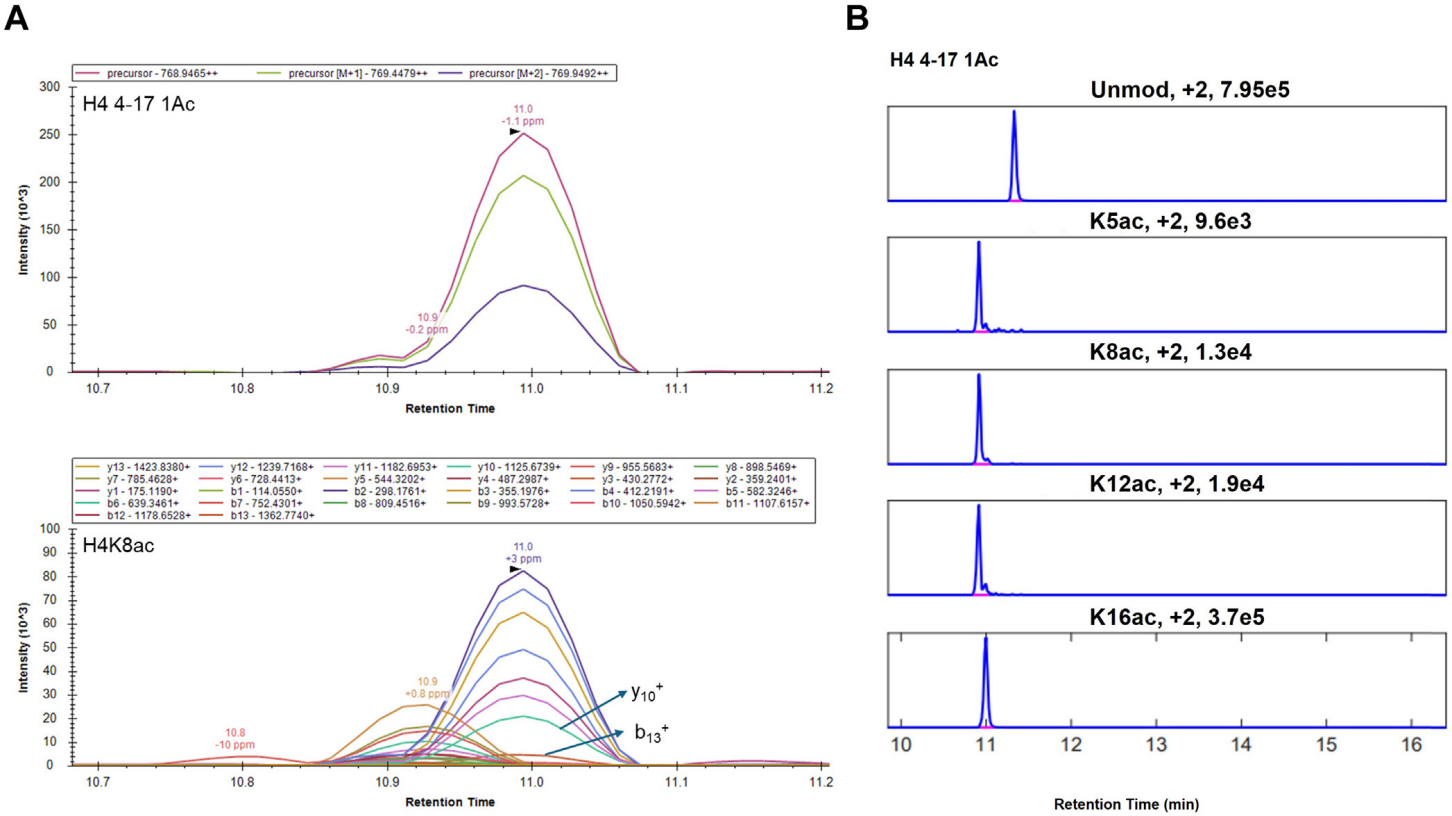
**Accurate quantification of histone peptides in ultrafast gradients using an updated version of EpiProfile.***A*, *top*, doubly charged H4 4–17 peptide bearing a single acetylation. *Bottom*, fragment ions produced by H4K8ac. Labeled ions y_10_^+^ and b_13_^+^ are useful for distinguishing peptide isomers. Data were visualized using Skyline. *B*, elution profiles and quantification of histone H4 4–17 peptides bearing a single acetylation at distinct sites by EpiProfile.

As the example shown in Figure 3, our complete data analysis workflow applies linear equations corresponding to the peak heights of distinct fragment ions situated between two sites of modification within the MS2 spectra, facilitating the accurate quantification of the isobaric histone peptides. Notably, the retention time is crucial to assigning peak identities for the software. The microflow system provided stable and reproducible retention time information comparable to results from the nanoflow system.

### Comparison of Measurements on the ORBITRAP EXPLORIS and SCIEX ZenoTOF: Large Global Histone Modifications Induced by HDACi Treatment

Histone acetylation‚ a reversible PTM‚ is catalyzed by histone acetyltransferases that transfer the acetyl moiety to lysine (K) residues leading to transcriptional activation. The removal of the acetyl group is mediated by HDAC enzymes. HDACis are potent epigenetic-modifying drugs and attractive for therapeutic use in treating various types of cancer, neurodegenerative, and cardiovascular diseases. These inhibitors can indirectly affect gene expression through crosstalk with DNA and histone methylation and cross-talk indirect modulation of other chromatin-associated protein activities (e.g., polycomb group and SWI–SNF [SWItch/Sucrose Non-Fermentable] complex [activity]) (62). The HDACi entinostat is an inhibitor of HDAC isoforms 1 and 3 (63). M344 is a selective HDAC6 inhibitor that is known to increase histone H3 and H4 acetylation (64). Treatment with each of these drugs is expected to produce large global changes in histone modifications.

**Fig. 4:**
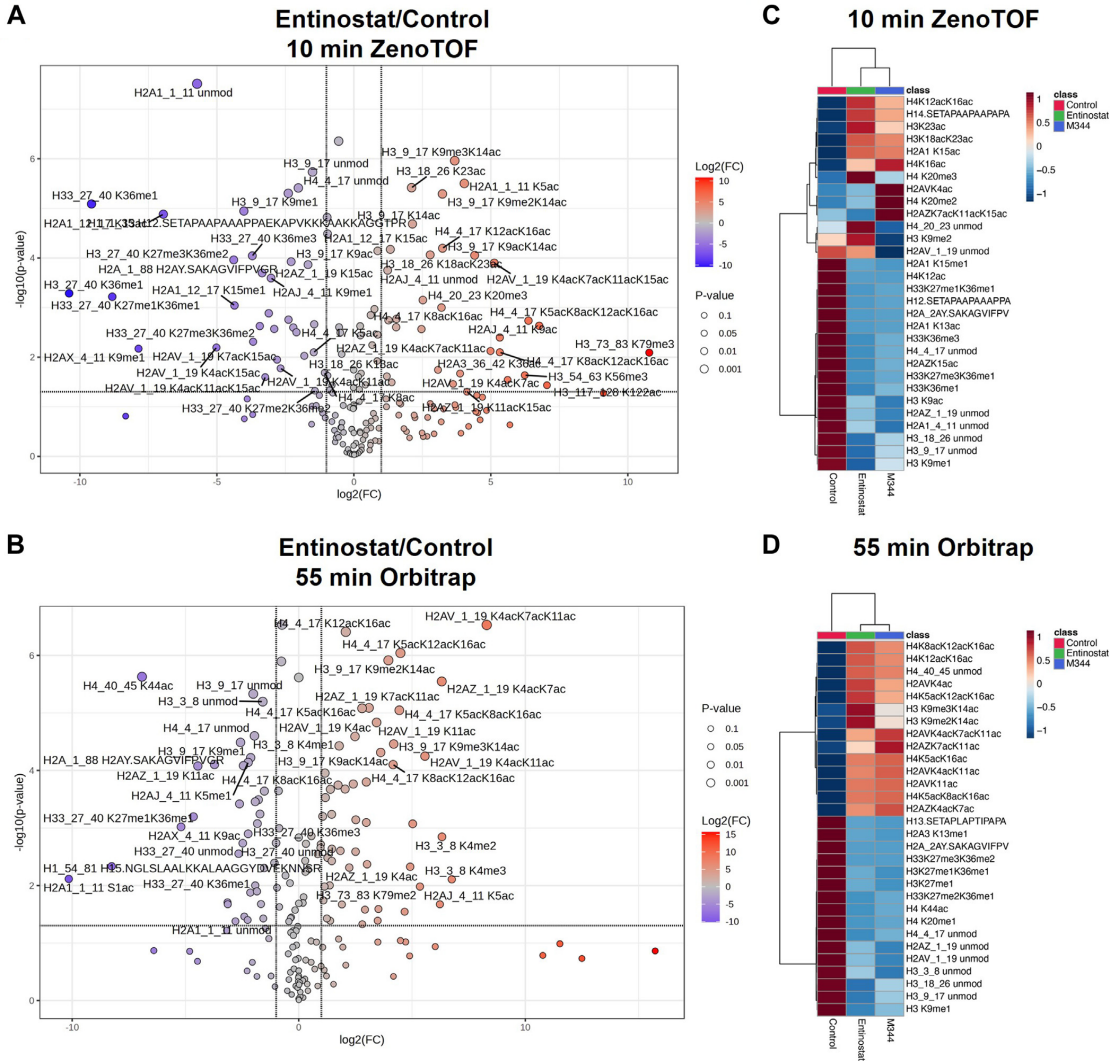
**Differentially expressed histone PTM marks for HDACi-treated samples.***A* and *B*, volcano plots illustrating differentially expressed histone PTM marks for entinostat–control samples observed using the 10-min and 55-min methods. Changes in log2 (entinostat/control) in PTM abundance on the *x*-axis are plotted against the significance level represented by *p* value on a log10 scale on the *y*-axis. The *dashed line* denotes a fold change of 2 in either direction, whereas the *horizontal line* indicates the significance threshold (*p* < 0.05). Metaboanalyst 6.0 was used for this plot. *C* and *D*, heatmaps generated through hierarchical clustering were used to compare control and samples with two treatment conditions, M344 and entinostat, targeting HDAC inhibition observed using either the 10-min gradient ZenoTOF method or the 55-min gradient Orbitrap method. Metabolanalyst 6.0 was used for this analysis. Study samples are shown on the horizontal axis, whereas the vertical axis represents global histone PTM marks. Control is denoted by *red square* treated with *blue* and entinostat-treated samples with *green*. The color spectrum from *dark blue* to *dark red* represents varying expression levels from low to high. HDACi, histone deacetylase inhibitor; PTM, post-translational modification.

In this study, we observed hyperacetylation of lysine residues in H3 and H4 histones induced by entinostat and M344 at specific sites, such as H3K18acK23ac, H3K14ac, H4K8acK12ac, and H4K12acK16ac‚ along with the acetylation of H2A variants as shown in volcano plots (Fig. 4, *A* and *B*). The top 30 most differentially expressed peptides were plotted in a heatmap for each method (Fig. 4, *C* and *D*). A similar trend for an increase in acetylation levels of histone H3 and H4, specifically H3K23ac, H3K14ac, H4K12acK16ac, and H2AZ K7acK11ac, was observed across both methods. The upregulation of H3K14ac, H3K18ac, and H4K12ac with similar fold changes was reported in the previous literature (65). Although entinostat-induced H2AV and H2AZ acetylation has not been previously reported, H2AZ acetylation has been shown to be increased by other HDACis (66). We were able to quantify not only the higher abundant H3 and H4 marks but also the lower level PTM marks such as H2AZ and H2AV acetylation marks using the 55-min gradient on the Exploris. These findings were well recapitulated in the 10-min ZenoTOF analysis. Observation of the robust and consistent effect of HDAC inhibition on histone modification is replicated well using a shorter gradient using one-third the instrument time compared with the Orbitrap method.

**Fig. 5:**
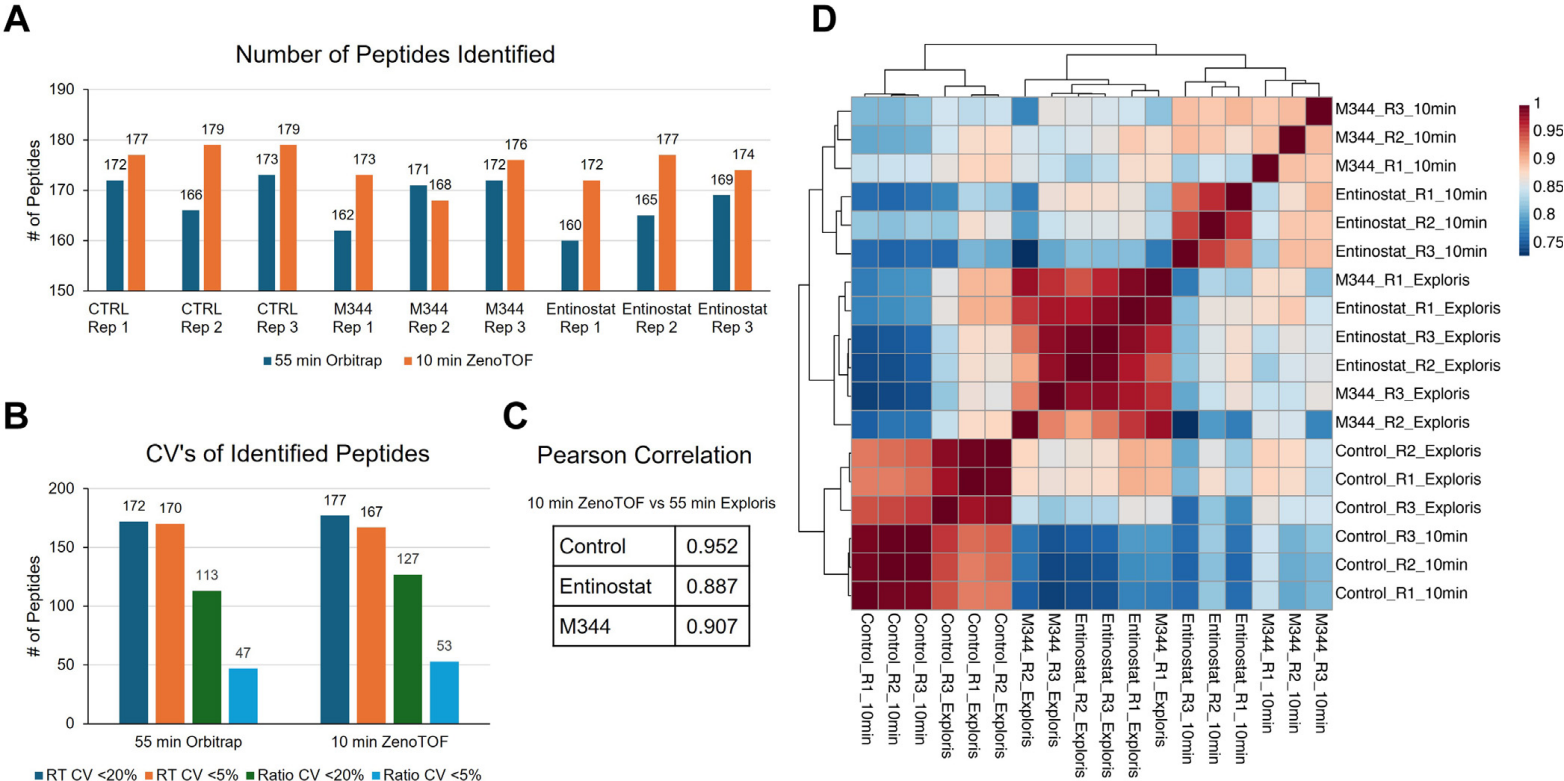
**Identification and quantification of histone peptides in HeLa cells by each method.***A*, the number of peptides identified in each sample (control, M344 treated, and entinostat treated) by each LC–MS method. *B*, correlation heatmap of all replicates of control, M344, and entinostat. *C*, the number of peptides identified by each method with a retention time CV below 20% and below 5%, and the number of peptides with peptide ratio CV below 20% and below 5% across all control replicates. *D*, correlation analysis of control, M344, and entinostat between the 10-min ZenoTOF method and 55-min Orbitrap method.

More than 150 histone peptides were identified in each replicate sample by each method. The 10-min ZenoTOF method matched or exceeded the number of peptide identifications achieved by the longer Orbitrap analysis in most cases despite the greatly reduced analysis time (Fig. 5*A*). In addition, histone peptide retention times and observed peptide ratios were consistent across runs for each method, as indicated in Figure 5*B*. Furthermore, to determine the variance levels between control and HDACi-treated samples from each method, we performed a Pearson correlation analysis (Fig. 5*C*). Replicates analyzed with each method correlate as shown in the correlation heatmap (Fig. 5*D*). The correlation coefficient between replicates of entinostat and M344 is less than 0.8, which is expected as these drugs have different effects on the histone global PTM landscape. This analysis revealed a high correlation between the results of the Orbitrap Exploris method and the ZenoTOF 10-min gradient method.

### Comparison of Measurements on the Orbitrap Exploris and Sciex ZenoTOF: Smaller Global Histone Modifications Occurring During the EMT

In cancer, EMT has been associated with the increased aggressiveness and metastasis of tumor cells. During the EMT process, alterations in the epigenetic landscape are involved to regulate transcription, resulting in the suppression of epithelial markers as well as the activation of mesenchymal markers. We asked if we could observe these epigenetic changes in EMT from the global profiling of histone PTMs, especially changes that were not large in fold change as induced by HDACi. We utilized an epithelial lung adenocarcinoma cell line, NCI-H1437, as a model to compare the histone PTM levels with or without induced EMT.

**Fig. 6:**
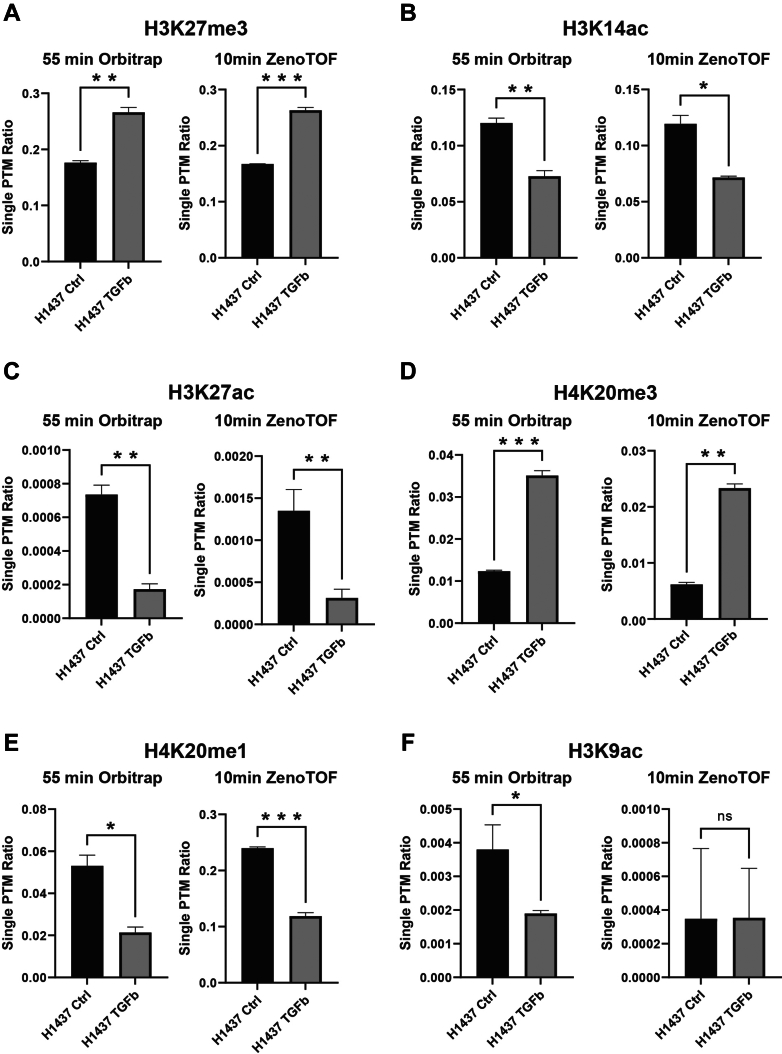
**Altered histone PTM marks in TGF-β-treated samples.** Calculated ratios for single histone PTMs as observed using either the previous 55-min Orbitrap Exploris method or the 10-min ZenoTOF 7600 method. *A* and *B*, the ZenoTOF method recapitulates both the significant difference in expression and the peptide ratio of relatively abundant marks H3K27me3 and H3K14ac. *C*–*E*, changes in the ratios of less abundant histone PTM marks H3K27ac, H4K20me3, and H4K20me1 can be observed using the 10-min ZenoTOF method. *F*, changes in the level of H3K9ac, an extremely low-level mark with an isomer complicating identification and quantification, are not consistently observed across the two methods. PTM, post-translational modification; TGF-β, transforming growth factor beta.

With a cutoff of 1.5 fold change and *p* value of 0.05, six marks were found significantly altered in TGF-β-treated samples by analysis on the Orbitrap Exploris using the previous 55 min gradient method. In the 10-min ZenoTOF analysis, significant changes in five of the six marks have been reproduced in only one-third of the instrument time (Fig. 6, *A*–*F*). This includes a rare mark H3K27ac (less than 0.1% present) (Fig. 6*C*). No additional significant change was observed. Taken together, the only different observation between the two LC–MS approaches from the same set of samples is the H3K9ac (Fig. 6*F*). This could potentially stem from the challenge of resolving the peptide containing this mark from the isomer containing H3K14ac. For the PTMs with higher abundance (H3K14ac and H3K27me3), the 10-min ZenoTOF analysis not only reported the same changes between conditions as the original Orbitrap method, but it also reported very close PTM ratios. For more rare marks (H3K27ac, H4K20me, and H4K20me3), there are variations in absolute PTM ratios reported by the two methods; however, the same trend of changes between conditions was still consistent.

**Fig. 7:**
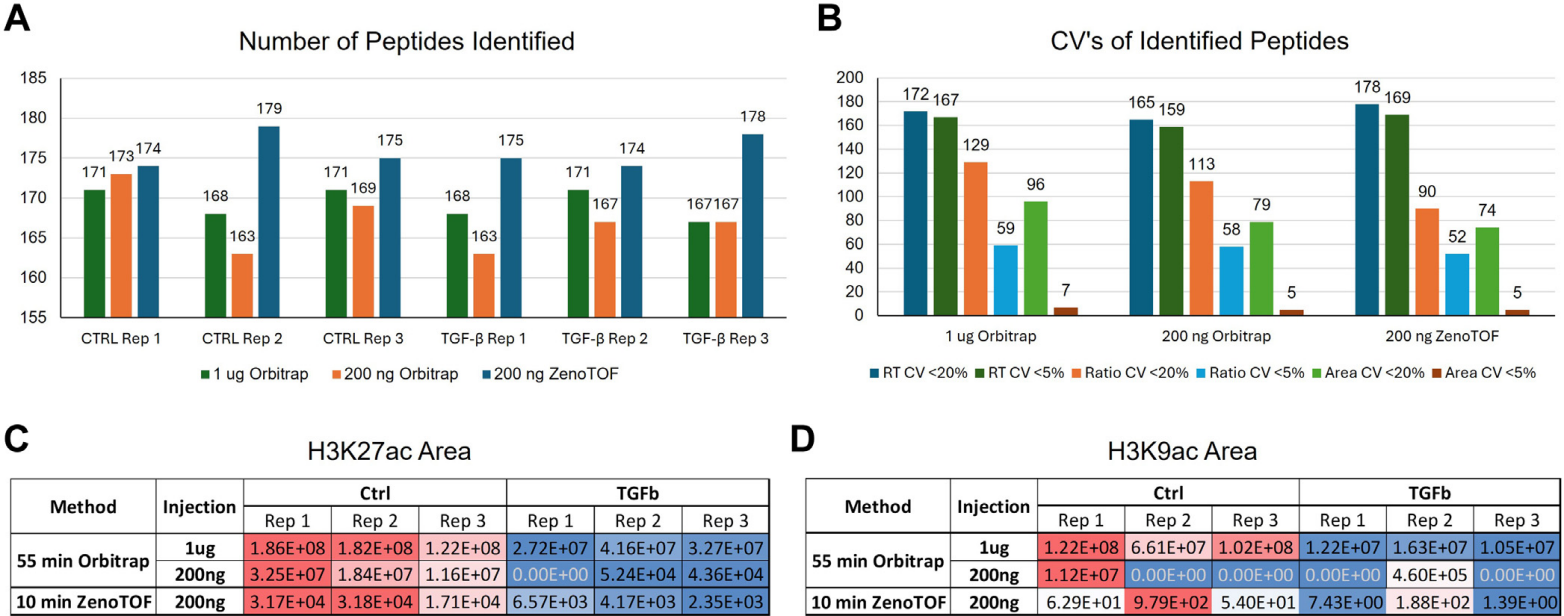
**Identifications by injection amount and LC–MS method.***A*, number of peptides identified in each control and TGF-β treated biological replicate by injection of either 200 ng or 1 μg on the Orbitrap and 200 ng on the ZenoTOF. *B*, number of peptides identified in control samples with peptide ratio, area, and retention time CVs below 5% and below 20% for each method and injection amount. *C*, area of H3K27ac peptide observed in each control and TGF-β treated sample by method and injection amount. *D*, area of H3K9ac peptide observed in each sample by method and injection amount. TGF-β, transforming growth factor beta.

The previous method was developed on a first-generation Thermo Orbitrap Q-Exactive and used 1 μg of histone sample for each analysis. To test whether this amount of sample is necessary for analysis on the Orbitrap Exploris, we injected 200 ng of each sample on the Exploris for comparison to the 1 μg Exploris and the 200 ng ZenoTOF data. Although injecting 200 ng on the Exploris results in a similar number of identified peptides and CV% values for retention time, peptide ratios, and areas comparable to the 1 μg injections (Fig. 7, *A* and *B*), the ZenoTOF enables better detection of low-level histone PTM marks using the same reduced sample amount. Some low-level histone PTM marks observed in both the ZenoTOF runs and the 1 μg Exploris runs are not reliably detected using 200 ng on the Exploris. For example, the H3K27ac peptide, which was significantly reduced in treated samples in both the 200 ng ZenoTOF and 1 μg Exploris data, is not detected in one of the treated samples using the smaller sample amount on the Exploris (Fig. 7*C*). Similarly, the H3K9ac peptide is not detected in most samples despite being detected in each sample using either 200 ng ZenoTOF or 1 μg on the Exploris (Fig. 7*D*).

### Analysis of 100 Injections of HeLa Histone Peptides in Less Than 2 Days on the Sciex ZenoTOF

**Fig. 8:**
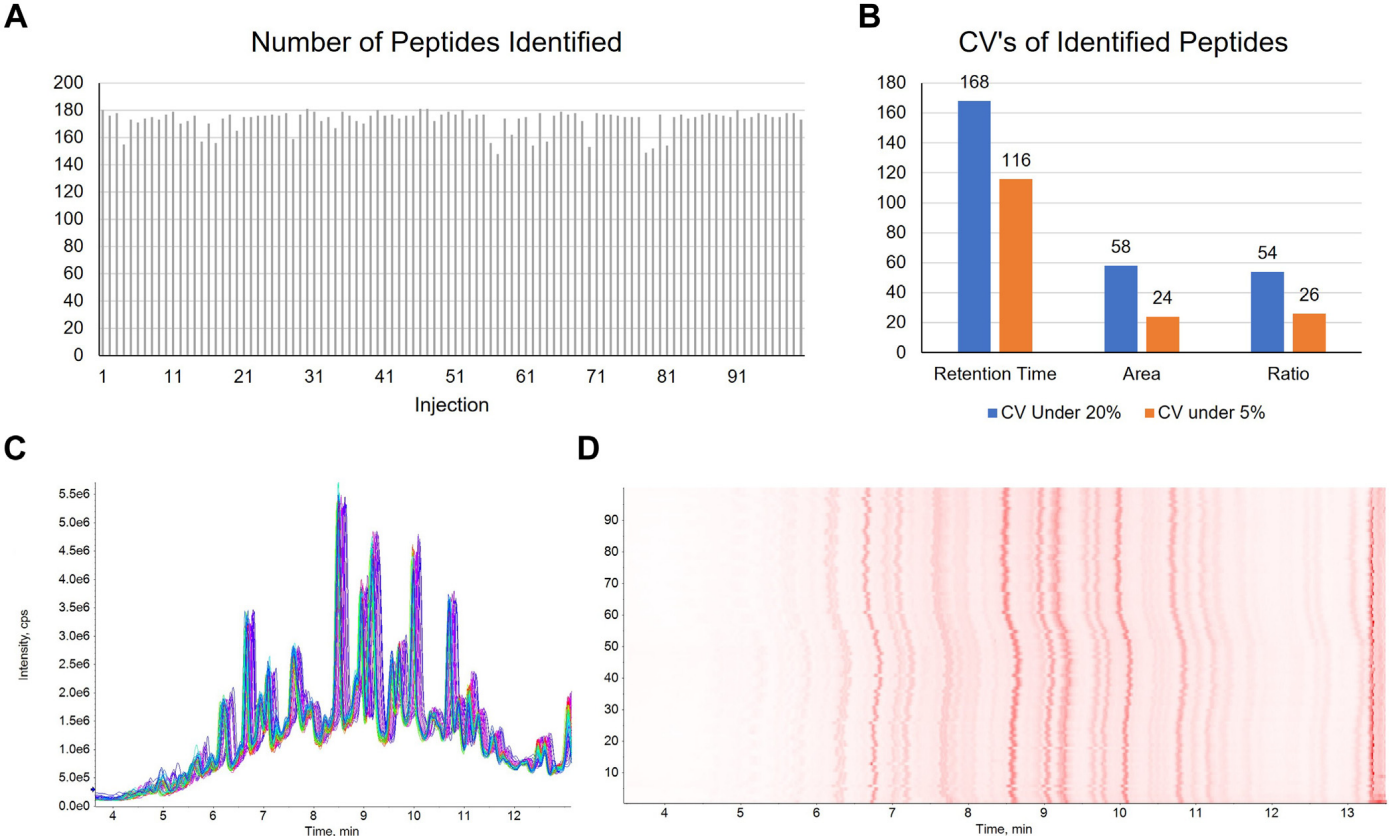
**About 100 LC–MS analyses of a histone peptide sample in <2 days.***A*, total number of peptides identified in each run. *B*, number of peptides with a CV below 20% and below 5% as calculated using observed retention times, peak areas, or peptide ratios. *C*, overlaid TIC for 50 consecutive injections. *D*, heat map chromatogram for TIC of all 100 runs. TIC, total ion chromatogram.

To demonstrate the speed and consistency of the method, a single sample of histone peptides extracted from HeLa cells was analyzed using the 10-min gradient ZenoSWATH DIA method 100 times in less than 2 days. The number of histone peptides identified in individual runs ranged from 148 to 181 (Fig. 8*A*). Peptide retention time stability was comparable to what was observed using both the nanoflow and microflow methods over shorter amounts of time and number of injections. Across the 2 days of runs, 168 peptides had a retention time CV% below 20 (Fig. 8*B*), which is similar to the results for TGF-β-treated samples and HDACi-treated samples described in Figures 5*B* and 7*B*. Observed peak areas and peptide ratios also showed low variation across runs (Fig. 8*B*). Chromatography and sample signal were consistent throughout the experiment, as seen in the overlaid total ion chromatograms and heat map chromatogram presented in Figure 8, *C* and *D*. The results demonstrate that the method is extremely high throughput and achieves robust and consistent identification and quantification of histone peptides while requiring very little (200 ng) sample and very short (>20 min) total run times.

## Conclusion

This work describes the development of a high-throughput LC–MS platform for quantifying the complex histone PTMs. We developed a novel ultrafast microflow method using a Sciex ZenoTOF 7600, which offers faster scan speeds and increased sensitivity, resulting in a reduction of run time to only 10 min per injection, with minimal sample amount (200 ng) required. This study compares the new method to a previous Orbitrap-based system, demonstrating that it achieves similar, if not improved, performance in identifying and quantifying hundreds of histone peptides. Key to the method's success is the integration of variable window SWATH DIA, allowing for precise isolation and identification of modified histone peptides across biological samples.

The results showed that histones extracted from cells treated with HDACis or TGF-β1 were accurately analyzed with this new platform, matching the performance of the previous method while using much less of the instrument time because of the short gradient. Furthermore, we introduced new computational tools, such as EpiProfile 2.2 and EpiConverter, to handle the new high-throughput data, facilitating large-scale studies of histone PTM landscapes. This method allows for fast and reproducible analysis of hundreds of modified histone peptides, capable of analyzing three times more samples per day compared with previous approaches. The findings offer a significant advancement in epigenetic research by enabling high-throughput histone PTM profiling with enhanced precision and speed.

## Data availability

LC–MS data files are available on MassIVE (MSV000094695 and MSV000094696) and ProteomeXChange (PXD052028 and PXD052029) for Thermo and SCIEX data, respectively. Annotated spectra are available on Panarama Public under the ProteomeXchange ID PXD056434. EpiProfile 2.2 is available on GitHub at github.com/zfyuan/EpiProfile2.0_Family. EpiConverter is available on GitHub at github.com/ChenfengZhao/EpiConverter.

## Dedications

It is an honor to contribute my lab’s research work for this special Molecular and Cellular Proteomics issue for Donald F. Hunt. I was encouraged to join Don’s lab by two prominent scientists I met when I was an undergraduate student, Jack Beauchamp (Caltech) and John Yates (Scripps). Both told me that if I wanted to study proteins by mass spectrometry, there was only one place to go to graduate school, so I headed east for Charlottesville in 2001. My time in Don’s lab was filled with what I feel was the most technical and even biological knowledge I have ever been exposed to in a short period of my career. The environment was vibrant, with many amazing people who would go on to make their own marks in science in the future such as Josh Coon, John Syka, Annie Evans, Sahana Mollah, and Beatrix Ueberheide just to name a few. Technology innovation and biochemical discoveries were happening on practically a weekly basis; it was exciting and inspiring beyond compare.

At the time I had arrived in Don’s lab, he was already collaborating with the late David Allis, applying mass spectrometry (MS) to histone and chromatin protein post-translational modifications (PTMs), an emerging field called epigenetics. The combination of labs moved quickly, and several high-profile manuscripts resulted. This not only advanced this new biological field but cemented the role of MS as an indispensable tool for chromatin biology and epigenetics research. Don solved many early challenges in the analysis of histone modifications, one of which was to introduce chemical derivatization (propionylation) in the bottom–up histone PTM MS analysis workflow. This derivatization works by modifying lysine residues with a propionyl group, so that when digested with trypsin, only arginine residues are cleaved. The resultant propionylated histone peptides are also more hydrophobic and contain less charge, which allows them to better retain on C18 columns, and to be easily fragmented by collisionally activated methods to provide more interpretable tandem mass spectra.

In my independent lab over my career, my research group has continued the methodology advances for enhanced characterization of modified histone peptides. Here, we describe our latest efforts to evolve our high-throughput bottom–up MS histone PTM analysis platform. The histone PTM field would not be the same without Don Hunt, and we are pleased we can contribute to this special issue to honor Don’s pioneering and impactful scientific career.

## Supplemental data

This article contains supplemental data.

## Conflict of interest

The authors declare no competing interests.

## Supplementary data

Supplemental Data Revised
