# Supplementary material for: Development of a High-Throughput Platform for Quantitation of Histone Modifications on a New QTOF Instrument
Source: Mol Cell Proteomics. 2024 Dec 19;24(1):100897. doi: 10.1016/j.mcpro.2024.100897 (PMC11787651; doi:10.1016/j.mcpro.2024.100897)
Supplement: Supplemental Data Revised [file mmc1.pdf]

Development of a high-throughput platform for quantitation of histone modifications on a new QTOF instrument

Emily Zahn[1], Yixuan Xie[1,2], Xingyu Liu[1], Rashmi Karki[1], Richard M. Searfoss[1], Francisca N. de Luna Vitorino[1], Joanna K. Lempiäinen[1], Joanna Gongora[1], Zongtao Lin[1], Chenfeng Zhao[3], Zuo-Fei Yuan[4], and Benjamin A. Garcia[1*]

[1]Department of Biochemistry and Molecular Biophysics, Washington University School of Medicine, St. Louis, Missouri 63110, United States
[2]State Key Laboratory of Genetic Engineering, Greater Bay Area Institute of Precision Medicine (Guangzhou), School of Life Sciences and Institutes of Biomedical Sciences, Fudan University, Shanghai, China
[3]Department of Computer Science and Engineering, Washington University in St. Louis, St. Louis, MO 63130, United States
[4]Center for Proteomics and Metabolomics, St. Jude Children's Research Hospital, Memphis, TN 38105, United States
*Corresponding author. Email: bagarcia@wustl.edu

Table S1	SWATH windows and detailed parameters used on ZenoTOF 7600.
Table S2	DIA windows used on Orbitrap Exploris 240.
Table S3	5-minute LC gradient used on M5 MicroLC.
Table S4	10-minute LC gradient used on M5 MicroLC.
Table S5	10-minute LC gradient used on ACQUITY UPLC M-Class.
Table S6	55-minute LC gradient used on Neo Vanquish UHPLC.
Table S7	EpiProfile output for Exploris HDACi runs.
Table S8	EpiProfile output for Exploris TGF-β 1 µg injections.
Table S9	EpiProfile output for Exploris TGF-β 200 ng injections.
Table S10	EpiProfile output for ZenoTOF HDACi 10 min method.
Table S11	EpiProfile output for ZenoTOF TGF-β 10 min method.
Table S12	EpiProfile output for ZenoTOF TGF-β 5 min method.
Table S13	EpiProfile output for ZenoTOF 100 injections of HeLa histone sample.
Table S14	Protein, modified peptide sequence, precursor, and product ion information.

Precursor ion start mass (Da)	Precursor ion stop mass (Da)	Declustering potential (V)	DP spread (V)	Collision energy (V)	CE spread (V)
299.5	304.5	80	0	14	0
303.5	310	80	0	14	0
309	317.6	80	0	14	0
316.6	329.8	80	0	15	0
328.8	341.4	80	0	15	0
340.4	350.9	80	0	16	0
349.9	356.7	80	0	16	0
355.7	362.5	80	0	16	0
361.5	368	80	0	17	0
367	373.8	80	0	17	0
372.8	380.2	80	0	17	0
379.2	387.5	80	0	18	0
386.5	396.3	80	0	18	0
395.3	418	80	0	19	0
417	430.2	80	0	20	0
429.2	438.7	80	0	20	0
437.7	447	80	0	21	0
446	455.2	80	0	21	0
454.2	464.7	80	0	21	0
463.7	479.3	80	0	22	0
478.3	490	80	0	23	0
489	499.1	80	0	23	0
498.1	506.1	80	0	23	0
505.1	512.5	80	0	24	0
511.5	519.3	80	0	24	0
518.3	526.6	80	0	24	0
525.6	537.6	80	0	25	0
536.6	553.4	80	0	25	0
552.4	565.3	80	0	26	0
564.3	575.4	80	0	27	0
574.4	585.4	80	0	27	0
584.4	595.8	80	0	28	0
594.8	617.5	80	0	28	0
616.5	671.1	80	0	30	0
670.1	721.8	80	0	32	0
720.8	765.7	80	0	35	0
764.7	781.9	80	0	37	0
780.9	796.8	80	0	37	0
795.8	816.9	80	0	38	0
815.9	896.2	80	0	40	0
895.2	950	80	0	43	0
949	1000	80	0	46	0

Table S1. SWATH windows and detailed parameters used on ZenoTOF 7600.

**Center Mass (*m/z*)**	**Window Width (*m/z*)**
307	24
330	24
353	24
376	24
399	24
422	24
445	24
468	24
491	24
514	24
537	24
560	24
583	24
606	24
629	24
652	24
675	24
698	24
721	24
744	24
767	24
790	24
813	24
836	24
859	24
882	24
905	24
928	24
951	24
974	24
997	24
1020	24
1043	24
1066	24
1089	24

Table S2. DIA windows used on Orbitrap Exploris 240.

Time (min)	Flowrate (µL/min)	%B	%A	Event
0.00	10.00	3.00	97.00	
1.00	10.00	3.00	97.00	Valve Load
2.00	10.00	5.00	95.00	
7.00	10.00	30.00	70.00	
7.50	10.00	80.00	20.00	
9.00	10.00	80.00	20.00	
9.50	10.00	3.00	97.00	
11.00	10.00	3.00	97.00	

Table S3. 5-minute LC gradient used on M5 MicroLC.

Time (min)	Flowrate (µL/min)	%B	%A	Event
0.00	10.00	3.00	97.00	
1.00	10.00	3.00	97.00	Valve Load
2.00	10.00	5.00	95.00	
12.00	10.00	32.00	68.00	
12.50	10.00	80.00	20.00	
14.00	10.00	80.00	20.00	
14.50	10.00	3.00	97.00	
18.00	10.00	3.00	97.00	

Table S4. 10-minute LC gradient used on M5 MicroLC.

Time (min)	Flowrate (µL/min)	%B	%A
0.00	10.00	3.00	97.00
1.00	10.00	3.00	97.00
11.00	10.00	32.00	68.00
12.00	10.00	80.00	20.00
14.00	10.00	80.00	20.00
15.00	10.00	3.00	97.00
20.00	10.00	3.00	97.00

Table S5. 10-minute LC gradient used on ACQUITY UPLC M-Class.

Time (min)	Duration (min)	Flowrate (µL/min)	%B	Volume (µL)	No. of Column Volumes
0.000	0.000	0.300	2.0	0.00	0
48.000	48.000	0.300	32.0	14.40	32.43
55.000	7.000	0.300	42.0	2.10	4.73
60.000	5.000	0.600	98.0	2.25	5.07

Table S6. 55-minute LC gradient used on Neo Vanquish UHPLC.
